# The influence of herbal medicine on serum motilin and its effect on human and animal model: a systematic review

**DOI:** 10.3389/fphar.2023.1286333

**Published:** 2023-12-14

**Authors:** Min-Seok Cho, Jae-Woo Park, Jinsung Kim, Seok-Jae Ko

**Affiliations:** ^1^ Department of Clinical Korean Medicine, Graduate School of Kyung Hee University, Seoul, Republic of Korea; ^2^ Department of Internal Korean Medicine, Kyung Hee University Hospital at Gangdong, Seoul, Republic of Korea; ^3^ Department of Gastroenterology, College of Korean Medicine, Kyung Hee University, Seoul, Republic of Korea

**Keywords:** motilin, herbal medicine, gastrointestinal motility, gastrointestinal tract, systematic review

## Abstract

**Introduction:** Motilin (MLN) is a gastrointestinal (GI) hormone produced in the upper small intestine. Its most well understood function is to participate in Phase III of the migrating myoelectric complex component of GI motility. Changes in MLN availability are associated with GI diseases such as gastroesophageal reflux disease and functional dyspepsia. Furthermore, herbal medicines have been used for several years to treat various GI disorders. We systematically reviewed clinical and animal studies on how herbal medicine affects the modulation of MLN and subsequently brings the therapeutic effects mainly focused on GI function.

**Methods:** We searched the PubMed, Embase, Cochrane, and Web of Science databases to collect all articles published until 30 July 2023, that reported the measurement of plasma MLN levels in human randomized controlled trials and *in vivo* herbal medicine studies. The collected characteristics of the articles included the name and ingredients of the herbal medicine, physiological and symptomatic changes after administering the herbal medicine, changes in plasma MLN levels, key findings, and mechanisms of action. The frequency patterns (FPs) of botanical drug use and their correlations were investigated using an FP growth algorithm.

**Results:** Nine clinical studies with 1,308 participants and 20 animal studies were included in the final analyses. Herbal medicines in clinical studies have shown therapeutic effects in association with increased levels of MLN, including GI motility regulation and symptom improvement. Herbal medicines have also shown anti-stress, anti-tumor, and anti-inflammatory effects *in vivo*. Various biochemical markers may correlate with MLN levels. Markers may have a positive correlation with plasma MLN levels included ghrelin, acetylcholine, and secretin, whereas a negative correlation included triglycerides and prostaglandin E_2_. Markers, such as gastrin and somatostatin, did not show any correlation with plasma MLN levels. Based on the FP growth algorithm, *Glycyrrhiza uralensis* and *Paeonia japonica* were the most frequently used species.

**Conclusion:** Herbal medicine may have therapeutic effects mainly on GI symptoms with involvement of MLN regulation and may be considered as an alternative option for the treatment of GI diseases. Further studies with more solid evidence are needed to confirm the efficacy and mechanisms of action of herbal medicines.

**Systematic Review Registration:** https://www.crd.york.ac.uk/prospero/display_record.php?RecordID=443244, identifier CRD42023443244.

## 1 Introduction

Motilin (MLN) is a gastric peptide hormone that was first isolated in the early 1970s and is known to control gastrointestinal (GI) tract movement (Brown, 1967; Brown et al., 1972). M cells, which prevail in the proximal region of the duodenum, secrete MLN in humans (Walsh et al., 1994). Notably, increased serum MLN levels accelerate bowel movements, and MLN exerts its effects by binding to MLN receptors (Itoh, 1997). Erythromycin (ER) was the first known MLN receptor agonist (Feighner et al., 1999) and was first used as a macrolide antibiotic in 1952. Its side effects include vomiting and diarrhea, which are two of the major effects of MLN in the GI tract (Putzi et al., 1983; Berthet et al., 2010). In one study, researchers found that ER mimics the effects of MLN on GI contractions in dogs (Itoh et al., 1984). Subsequent studies have supported the prokinetic activity of ER (Annese et al., 1992). Since it is known that MLN receptor agonists, such as ER, can target GI motility disorders, there have been numerous trials to create or identify other MLN receptor agonists; however, none of them have been successful either clinically or commercially **(** Omura et al., 1987; Tsuzuki et al., 1989 **)**. For example, the effect of the motilide ABT-229, an MLN receptor agonist, was assessed in randomized controlled trials (RCTs) involving 612 patients with functional dyspepsia, but it failed to provide symptomatic relief in patients with delayed gastric emptying (Talley et al., 2000). This disappointing outcome was attributed to receptor desensitization, which caused the receptor to react less strongly to the ligand (Tack and Peeters, 2001), due in part to the use of an inappropriate dosing regime and potential non-selectivity of action (Sanger et al., 2013).

Herbal formulas have been used as alternatives to Western medicine for treating GI symptoms such as constipation, diarrhea, and dyspepsia (Zhang et al., 2013; Zhang and Guo, 2015; Ren et al., 2021). Furthermore, several herbal medicines have been reported to affect plasma MLN levels. *Daikenchuto*, one of Japan’s most frequently prescribed traditional medicines, increases plasma MLN levels, enhances GI motility, and improves gastric dysrhythmia and postoperative gastroparesis (Mochiki et al., 2010). Notably, previous systematic reviews have reported that *Rikkunshito* and *Banxia-xiexin tang*, which are traditional Asian herbal medicines, are effective at improving the symptoms of functional dyspepsia by promoting MLN secretion (Ko et al., 2021; Kim et al., 2023).

Here, we conducted a systematic review to investigate the influence of herbal medicine on serum MLN and its effect on human and animal model. We assessed the effect of herbal medicine on the various symptoms including GI symptoms, and the direct or indirect correlation between symptoms and changes in MLN. The possible relationship between biochemical findings and plasma MLN was also investigated. In addition, we checked the most used botanical drugs and their combinations that increased or lowered plasma MLN using an association rule algorithm.

## 2 Methods

### 2.1 Objectives and registration

The objectives of this review were (1) to systematically review RCTs and *in vivo* studies investigating the effects of herbal medicine on various disorders mainly focused on GI function by regulating serum MLN levels and (2) to elucidate the mechanisms responsible for the change in GI function by MLN after the administration of herbal medicine. This systematic review followed the Preferred Reporting Items for Systematic Reviews and Meta-Analysis 2020 guidelines (Page et al., 2021) and provided its checklist as a Supplementary Material S1. This systematic review was registered in The International Prospective Register of Systematic Reviews under the identifier CRD42023443244.

### 2.2 Inclusion and exclusion criteria

This systematic review included RCTs and *in vivo* studies. *In vitro* studies, case reports, case series, and reviews were excluded. Studies that evaluated the effects of herbal medicines on serum MLN levels were included. To be eligible for inclusion, the herbal medicines had to consist of multiple botanical drugs (two minimum), but they could be administered in various forms such as decoctions, powders, granules, pills, and capsules. Additionally, patented drugs or over-the-counter drugs composed of botanical drugs, such as *Dalitong* granules (Nanchang Hongyi Pharmaceutical Co., Ltd., Shanghai, China), were included. Studies combining traditional therapies other than herbal medicines, such as acupuncture, moxibustion, and cupping therapy, were excluded.

### 2.3 Search strategy

RCTs and *in vivo* studies were searched separately.

#### 2.3.1 Search strategy for RCTs

Patients who received herbal medicine as an intervention and whose serum MLN levels were measured were included. All types of dosage forms of oral herbal medicines were used as keywords. Data were extracted from PubMed, Embase, Web of Science, and Cochrane Library databases until July 2023. The search strategy for RCTs is shown in Table 1.

**TABLE 1:** Search strategy for randomized controlled trials in PubMed.

Search number	Search items
#1	(motilin[Mesh]) OR (motilin[TW])
#2	((randomized[TW]) OR (random*[TW])) OR (RCT*[TW])
#3	#1 and #2
#4	(“herb*"[All Fields] OR “formula*"[All Fields] OR “decoction*"[All Fields] OR “granule*"[All Fields] OR “pill*"[All Fields] OR “powder*"[All Fields] OR “capsule*"[All Fields] OR “solution*"[All Fields] OR “tang*"[All Fields] OR “prescription*"[All Fields])
#5	#3 and #4

#### 2.3.2 Search strategy for *in vivo* studies

The search keywords included all animals, such as rats, mice, dogs, rabbits, and monkeys, as well as MLN and different dosage forms of herbal medicine. Data were extracted from PubMed, Embase, Web of Science, and Cochrane until July 2023. The search strategy used for *in vivo* studies is shown in Table 2.

**TABLE 2:** Search strategy for *in vivo* studies in PubMed.

Search number	Search items
#1	“motilin"[MeSH Terms] OR “motilin"[All Fields] OR “motilin s"[All Fields] OR “motilins"[All Fields]
#2	“motilin"[MeSH Terms]
#3	#1 or #2
#4	herb*
#5	#3 and #4
#6	“rats"[MeSH Terms] OR “rats"[All Fields] OR “rat"[All Fields] OR “mice"[MeSH Terms] OR “mice"[All Fields] OR “mouse"[All Fields] OR “mouses"[All Fields] OR “rabbit*"[All Fields] OR “dogs"[MeSH Terms] OR “dogs"[All Fields] OR “dog"[All Fields] OR “monkey*"[All Fields] OR “animal*"[All Fields]
#7	“formula*"[All Fields] OR “decoction*"[All Fields] OR “granule*"[All Fields] OR “pill*"[All Fields] OR “powder*"[All Fields] OR “capsule*"[All Fields] OR “solution*"[All Fields] OR “tang*"[All Fields] OR “prescription*"[All Fields]
#8	#3 and (#4 or #7) and #6

### 2.4 Selection and data extraction

Two authors (M-SC and J-WP) independently screened the studies to assess their eligibility for inclusion. Eligibility was evaluated by sequentially screening the articles’ titles, abstracts, and full texts. Endnote X9 (Clarivate Analytics, Philadelphia, PA, United States) was used to manage the search results. The independently extracted data from the studies was entered in a standard data extraction form. The form for the RCTs included information on the studies, such as the intervention, disease, sample size, treatment duration, change in plasma MLN levels, publication year, main outcome, and efficacy. The form for *in vivo* studies included information such as the intervention, animal breed, disease model, administration method, mechanisms, changes in MLN levels, main outcome, and efficacy. Discrepancies between the two authors (M-SC and J-WP) were resolved through discussion. If agreement was not reached, an arbiter (S-JK) intervened.

### 2.5 Quality assessment

Risk of bias evaluation was conducted for the RCTs. Review Manager (V5.3; The Nordic Cochrane Center, The Cochrane Collaboration, 2014; Copenhagen, Denmark) was used to manage the data. Two authors (M-SC and J-WP) independently assessed the risk of bias using the Cochrane Risk of Bias Tool (RoB 2) with the following items: (1) bias arising from the randomization process, (2) bias due to deviations from intended interventions, (3) bias due to missing outcome data, (4) bias in the measurement of the outcome, (5) bias in the selection of the reported results, and (6) overall bias. The results were categorized into three groups: low, high, or unclear risk of bias. All discrepancies between the two evaluators (M-SC and J-WP) were discussed. An arbiter (SK) intervened when needed.

### 2.6 Frequent pattern growth algorithm for data analysis

Frequent pattern (FP) growth algorithm analysis is a data-mining technique that has been widely used in healthcare, with the aim of discovering valuable correlations implicit in large data sets (Ait-Mlouk et al., 2017; Rauch, 2019). The FP growth algorithm produces frequent item sets by compressing them into an FP tree and retaining related information about the frequent items (Li et al., 2022). In recent years, the FP growth algorithm has been used in the field of herbal medicine and has achieved many gratifying research results (Leem et al., 2018; Chen et al., 2020). Therefore, an FP growth algorithm was used to determine the frequency patterns of botanical drug use and their correlations. After mining the botanical drugs used more than four times for all included studies, we listed them in order of frequency. Botanical drugs used in combination with other botanical drug were connected individually. Each node represents the items of botanical drug and the most frequently used item set was identified.

## 3 Results

### 3.1 Search process for included studies

#### 3.1.1 Searching and narrowing down RCT studies

**FIGURE 1:**
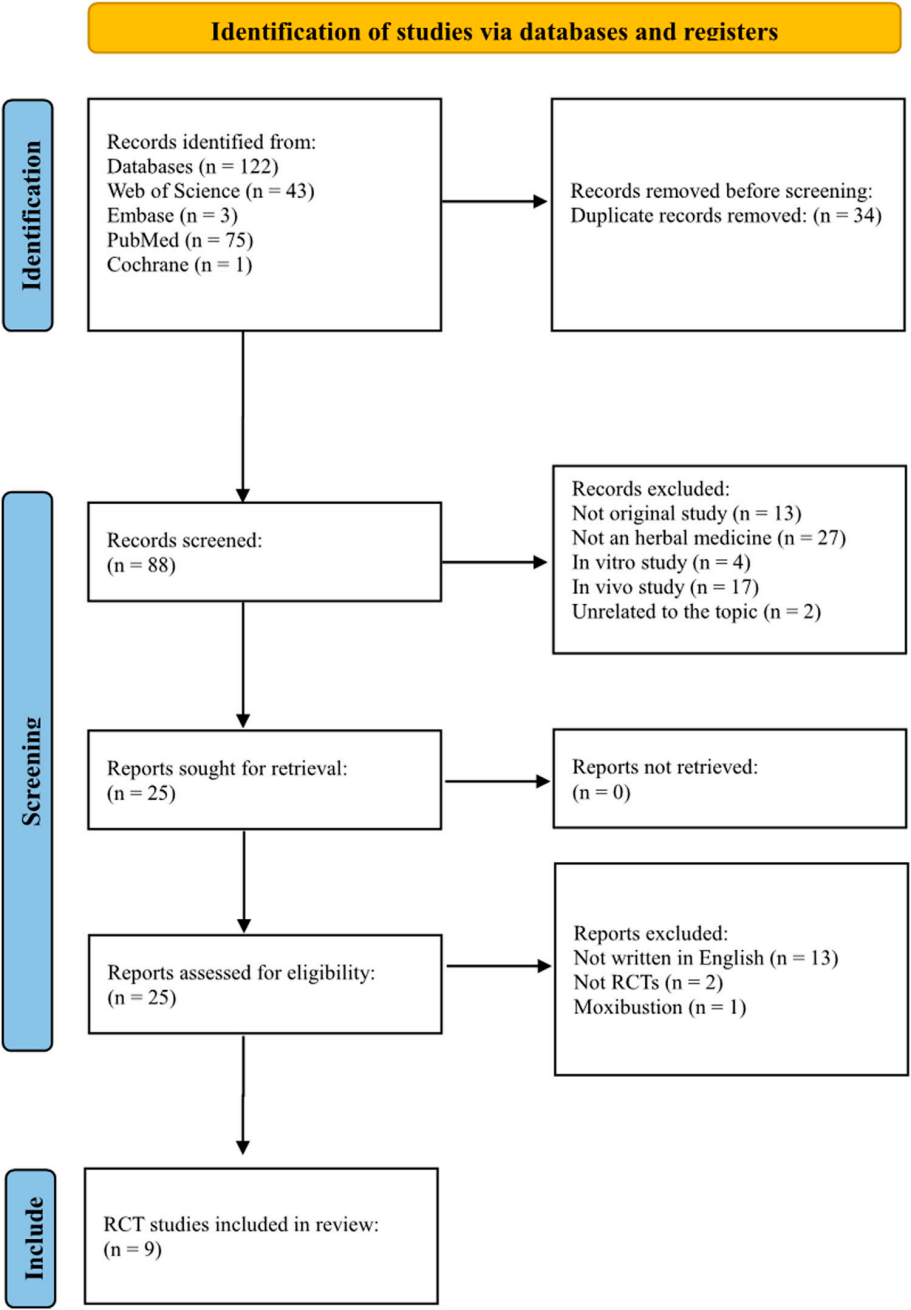
PRISMA flow diagram of the randomized controlled trials.

One hundred twenty-two records were identified in the database. Of the 122 studies, 34 were duplicates. Of the 88 remaining studies, 13 were not original studies, 27 did not use herbal medicine, four were *in vitro* studies, 17 were animal studies, and two were on unrelated topics. After reviewing 25 studies, nine were included in this review. This process is shown in Figure 1.

#### 3.1.2 Searching and narrowing down *in vivo* studies

**FIGURE 2:**
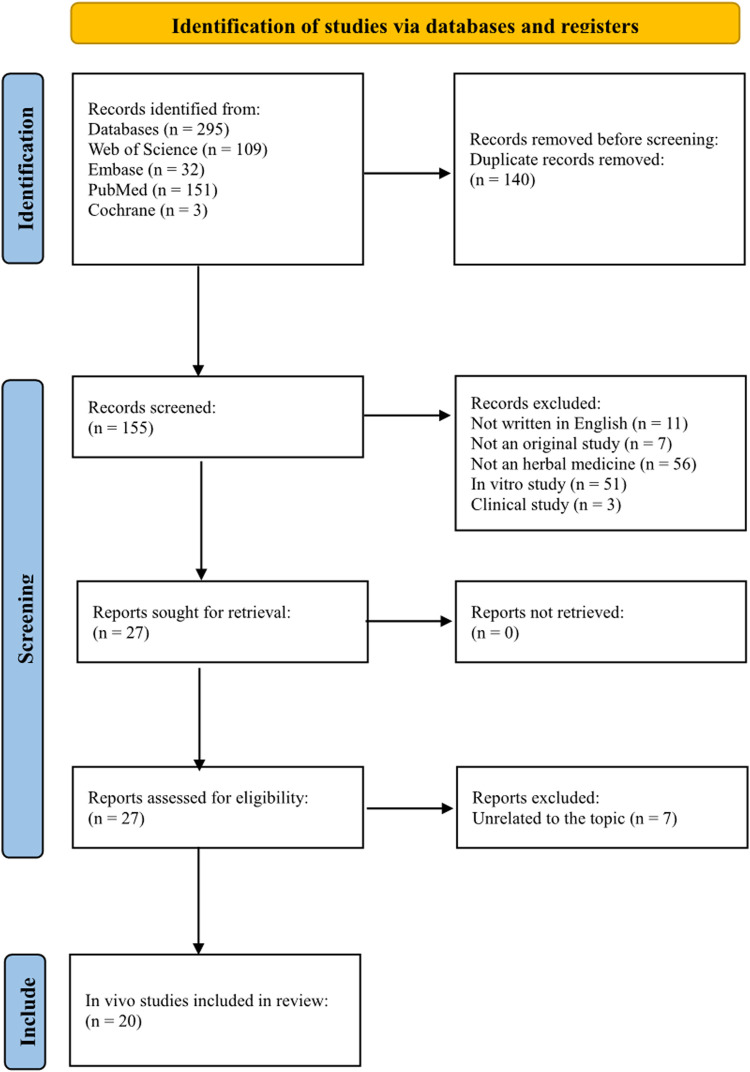
PRISMA flow diagram of the *in vivo* studies.

Two hundred ninety-five studies were identified from these databases. One hundred and forty duplicate records were removed. Of the 155 remaining studies, 11 were not written in English, seven were not original studies, 56 did not use herbal medicine, 51 were *in vitro* studies, and three were RCTs. After excluding these studies, 27 studies remained. Of the 27 studies, seven were on unrelated topics. Finally, 20 *in vivo* studies were included in this review (Figure 2).

### 3.2 Characteristics of included studies

#### 3.2.1 Description of the RCT studies

In eight of the nine studies, herbal medicines were used alone, and in the remaining study (Ren et al., 2021), herbal medicines and an antibiotic (cefuroxime) were used in combination. Seven studies were conducted on patients with GI diseases or symptoms, one included patients with psychiatric symptoms, and the remaining included healthy individuals. The number of patients varied from 33 to 635, and the positive control groups were placebo (five studies), a gastroprokinetic agent (two studies: mosapride), a health functional food (one study), and a combination of Western medicines (one study: probiotics, antiviral, and nonsteroidal anti-inflammatory drugs). Eight studies were conducted with two groups (treatment and control), whereas one study (Zhang and Guo, 2015) had four groups: one control group and three different treatment groups. In seven studies, herbal medicines increased serum MLN levels, whereas in two studies, herbal medicines decreased MLN levels. Notably, herbal medicines showed various therapeutic effects through increased MLN levels, including GI symptom improvement, an increase in duodenal and jejunal motility, and the alleviation of depression. Herbal medicines also shortened the treatment time for diarrhea in patients with upper respiratory tract infections and recovered GI function in patients after abdominal surgery through a decrease in MLN levels. The characteristics of the included RCTs are summarized in Table 3. Additional information on the herbal medicines used in the included RCTs, such as the extraction type, ingredients, and daily administration dose, is shown in Table 4. The value of MLN level, concentration unit and *p*-value are provided as Supplementary Material S2.

**TABLE 3:** Characteristics of the included randomized controlled trials.

Herbal medicine (treatment)	Patient inclusion criteria	Total (n) (treatment/control)	Positive control	Treatment period	Change in serum motilin levels	Primary outcome	Mechanisms	Efficacy	Adverse events (n) (treatment/control)	Reference
*Jianpiyangxue* granules	Patients with GI autonomic dysfunction	120 (60/60)	Vitamin B, oryzanol	4 weeks	↑	↑GAST, SS	↑IgG, IgM, IgA ↓CRP, IL-6	Improved GI autonomic dysfunction	(3/11)	Zhou and Wang (2021)
*Xingpi Yanger* granules with cefuroxime	Patients with upper respiratory tract infection with diarrhea	124 (62/62)	Ibuprofen suspension, ribavirin granules, *Bifidobacterium*, *Lactobacillus* tablets	1 week	↓	↑SS ↓GAST		Shortened treatment time	(4/13)	Ren et al. (2021)
*Xiangbin* prescription	Healthy volunteers	40 (30/10)	Placebo (licorice powder mix)	1 day	↑	↑GHRL		↑ Duodenal and jejunal motility	(0,0)	Jiang et al. (2017)
*XiangBin* granules	Patients with abdominal surgery	117 (79/38)	Placebo (dextrin 1,000 g)	1 week	↓ (1st day)	↓ Time until the first passage of flatus	↓CRH (1st day), VIP	Promoted the recovery of GI function	n/a	Wen et al. (2016)
*Dalitong* granules	Patients with functional dyspepsia (aged 17–69 years)	635 (158/160/^a^)	Mosapride	4 weeks (60th week, final checkup)	↑ (4th, 60th week)	↑Quality of life score ↓Symptom score		Alleviated dyspepsia symptoms	(0,0)	Zhang and Guo (2015)
Modified *Dachengqi Tang*	Patients with postoperative esophageal cancer	60 (30/30)	Placebo (normal saline)	3 d	↑	↓Time until the first flatus, time until the first defecation, time until the first intestinal sounds	↓VIP	↑GI motility	(1,0)	Xu et al. (2015)
*Xiaoyao* pill	Perimenopausal women with depression	180 (90/90)	Placebo (*Fructus setariae* germinates)	8 weeks	↑	↓HRSD score	↑GAST	↓Depression score	(0,5)	Du et al. (2014)
*Fuzhengliqi* mixture	Patients with functional constipation	560 (140/140/^a^)	Mosapride, macrogol 4,000	6 weeks (60th week, final checkup)	↑	↓Defecation interval, stool properties, constipation symptoms, accompanying symptoms, total symptoms		Improved functional constipation	(0,2)	Zhang et al. (2013)
*Da-Cheng-Qi-Tang*	Patients with abdominal surgery	33 (13/20)	Placebo (normal solution)	4 d	n/a	↑ Ratio of EGG normal frequency, the power of EGG (2nd, 3rd day), normal bowel peristalsis	↑The power of MMC III (1st, 2nd day in the proximal jejunum)	Improved GI function	n/a	Qi et al. (2007)
*Da-Cheng-Qi-Tang*	Patients with cholecystectomy	36 (21/15)	Placebo (normal solution)	4 d	↑ (1st, 2nd day)			Improved GI function	n/a	

EA: electroacupuncture; GI: gastrointestinal; GAST: gastrin; SS: somatostatin; Ig: immunoglobulin; CRP: C-reactive protein; IL: interleukin; GHRL: ghrelin; CRH: corticotropin releasing hormone; VIP: vasoactive intestinal peptide; HRSD: hamilton rating scale for depression; EGG: electrogastrography; MMC: migrating motor complex; n/a: not applicable; ↑: significant increase; ↓: significant decrease.

^a^ The two groups that did not use herbal medicines were excluded from the study.

**TABLE 4:** Information on herbal medicines used in the included randomized controlled trials.

Herbal medicine	Extraction	Ingredients	Daily dose	Reference
*Jianpiyangxue* granules	Water	*Codonopsis pilosula (Franch.) Nannf. [Campanulaceae; Codonopsis pilosula dried root] 15 g, Atractylodis macrocephalae, Atractylodes macrocephala Koidz. [Asteraceae; Atractylodes macrocephala dried rhizome], Ziziphus jujuba Mill. [Rhamnaceae; Ziziphus jujuba dried ripe fruit] 30 g, Poria cocos (Schw.) Wolf [Polyporaceae; Poria cocos sclerotium], Conioselinum anthriscoides ‘Chuanxiong’ [Apiaceae; Conioselinum anthriscoides dried rhizome] 20 g, Anemarrhena asphodeloides Bunge [Asparagaceae; Anemarrhena asphodeloides dried rhizome] 15 g, Schisandra chinensis (Turcz.) Baill. [Schisandraceae; Schisandra chinensis dried ripe fruit] 15 g, Reynoutria multiflora (Thunb.) Moldenke [Polygonaceae; Reynoutria multiflora dried lianoid stem] 20 g, and Glycyrrhiza glabra L. rhizome [Fabaceae; Glycyrrhiza glabra dried root and rhizome] 10 g*	150 mL, twice a day	Zhou and Wang (2021)
*Xingpi Yanger* granules	OTC	n/a	Children younger than 1 year:	Ren et al. (2021)
			2 g, twice per day; children aged 1–2 years:	
			4 g, twice per day; children aged 3–6 years:	
			4 g, 3 times per day	
*Xiangbin* prescription	Water	*Wurfbainia villosa* (Lour.) Škorničk. and A.D.Poulsen [Zingiberaceae; dried ripe fruit] fruit 6 g, *Lindera aggregata* (Sims) Kosterm. [*Lauraceae*; *Lindera aggregate* dried root tuber] 10 g, *Prunus persica* (L.) Batsch [Rosaceae; *Prunus persica* dried ripe seed] 10 g, *Panax ginseng* C.A.Mey. [*Araliaceae*; *Panax ginseng* dried root 9 g, and *Areca catechu* L. [*Arecaceae*; *Areca catechu* dried pericarp] 10 g.	200 mL concoction, once, 4 h later in the experiment	Jiang et al. (2017)
*XiangBin* granules	OTC, diluted to 50 mL per bag	*Areca catechu* L. [*Arecaceae*; *Areca catechu* dried pericarp], *Panax ginseng* C.A.Mey. [*Araliaceae*; *Panax ginseng* dried root], *Lindera aggregata* (Sims) Kosterm. [*Lauraceae*; *Lindera aggregate*, dried root tuber], *Aquilaria malaccensis* Lam. [*Thymelaeaceae*; *Aquilaria malaccensis* dried heartwood], and *Prunus persica* (L.) Batsch [*Rosaceae*; *Prunus persica* dried ripe seed]. Amount n/a.	50 mL per bag, twice a day (9 AM and 4 p.m.)	Wen et al. (2016)
*Dalitong* granules	OTC	n/a	6 g, 30 min before meals, 3 times daily	Zhang and Guo (2015)
Modified *Dachengqi Tang*	Water	*Rheum palmatum* L. [*Polygonaceae*; *Rheum palmatum* dried root and rhizome] 10 g, *Natrii sulfas* 5 g, *Magnolia officinalis* Rehder and E.H.Wilson [*Magnoliaceae*; *Magnolia officinalis* dried stem bark, root bark or branch bark] 15 g, *Citrus × aurantium* L. [*Rutaceae*; *Citrus × aurantium* dried, immature fruit] 15 g, *Angelica sinensis* (Oliv.) Diels [*Apiaceae*; *Angelica sinensis* dried root] 15 g, *Astragalus mongholicus* Bunge [*Fabaceae*; *Astragalus mongholicus* dried root] 15 g, *Paeonia lactiflora* Pall. [*Paeoniaceae*; *Paeonia lactiflora* dried root] 15 g, and *Lindera aggregata* (Sims) Kosterm. [*Lauraceae*; *Lindera aggregate* dried root tuber] 10 g.	150 mL, once a day on the morning of the first, second, and third day after surgery	Xu et al. (2015)
*Xiaoyao* pill	OTC	*Bupleurum falcatum* L. [*Apiaceae*; *Bupleurum falcatum* root], *Angelica sinensis* (Oliv.) Diels [*Apiaceae*; *Angelica sinensis* dried root], *Paeonia lactiflora* Pall. [*Paeoniaceae*; *Paeonia lactiflora* dried root]*,* roasted *Atractylodes macrocephala* Koidz. [*Asteraceae*; *Atractylodes macrocephala* dried rhizome], *Poria cocos* (Schw.) Wolf [Polyporaceae; *Poria cocos sclerotium*]*, Glycyrrhiza glabra* L. [*Fabaceae*; *Glycyrrhiza glabra* dried root and rhizome], *Mentha canadensis* L. [*Lamiaceae*’ *Mentha canadensis* dried aerial parts], and *Zingiber officinale* Roscoe [Zingiberaceae; *Zingiber officinale* dried rhizome]	3 g each time, 30 min before breakfast and supper, for 8 weeks	Du et al. (2014)
*Fuzhengliqi* mixture	n/a	n/a	60 mL, twice a day	Zhang et al. (2013)
*Da-Cheng-Qi-Tang*	Water	*Rheum palmatum* L. [Polygonaceae; *Rheum palmatum* dried root and rhizome] 12 g, *Magnolia officinalis* Rehder and E.H.Wilson [*Magnoliaceae*; *Magnolia officinalis* dried stem bark, root bark or branch bark] 9 g, *Citrus × aurantium* L. [*Rutaceae*; *Citrus × aurantium* dried, immature fruit] 9 g, and *Natrii sulfas* 9 g.	50 mL, unclear daily dosage	Qi et al. (2007)

OTC, over-the-counter medicine; n/a, not applicable.

#### 3.2.2 Description of the *in vivo* studies

Of the 20 studies, 12, five, two, and one were conducted in rats, mice, pigs, and dogs, respectively. In 19 studies, herbal medicine was used alone, while in the remaining study (Liu et al., 2020), herbal medicine and an antidiarrheal agent (diphenoxylate) were combined. Furthermore, in most studies (16 studies), herbal medicines increased serum MLN levels, whereas three studies reported a decrease, and one study found no significant change. Most studies (13 studies) observed a laxative effect or increased GI motility due to the herbal medicine. Other reported effects included anti-stress, anti-tumor, and anti-inflammatory effects, and liver and gastric mucosa protection. The characteristics of the included *in vivo* studies are summarized in Table 5, while additional details about the herbal medicines, such as the extraction type, ingredients, and daily dose, are described in Table 6. The value of MLN level, concentration unit and *p*-value are provided as Supplementary Material S2.

**TABLE 5:** Characteristics of the included *in vivo* studies.

Animal breed	Herbal medicine (treatment)	Disease model	Positive control	Administration method	Treatment duration	Mechanisms	Motilin	Main outcome	Efficacy	Reference
SD rats	*Fuzi Lizhong* pill	Spleen-Yang deficiency	n/a	n/a	15 d	↓MDA, IL-1α, IL-6	↓	↑Visceral index of spleen and kidney	Therapeutic effect on GI motility and digestive function	Z. Zhang et al., 2021
Wistar rats	Invigorating qi and hemostasis formula	Ischemia‒reperfusion	Clopidogrel pantoprazole	Intragastric	2 weeks	↓The platelet aggregation rate	↑	↑GAST, COX-1, PGE_2_	Decreasing platelet activation, anti-inflammatory effect	C. H. Zhang et al., 2021
KM mice	*Ciji-Hua’ai-Baosheng* II	Chemotherapy model	5-FU	Intraperitoneal	2 weeks	↑EGF, OXA, PGE_2_, SOD	↑	↑GAST, GHRL, NPY	Inhibitory effect on tumors	Xi et al. (2021)
						↓MDA, leptin				
SD rats	*Dashanzha* pill	Dyspepsia	Domperidone	Intragastric	2 weeks	↓GRP78, PERK, eIF2α	↑	↑GAST	Decreased endoplasmic reticulum stress, relief of dyspepsia	Liu et al. (2021)
								↓VIP		
SD rats	*Tiantian* capsule	Constipation	Hemp seed soft capsule	n/a	2 weeks	↑SP, c-kit	↑	↑Fecal pellet number, fecal water content, stomach emptying, GI transit (low dose)	Laxative effect	Li et al. (2021)
						↓SS, VIP, ET-1				
n/a (mice)	*BojungikkiTang*	Normal control	n/a	Intragastric	30 min	↑ITR, c-kit expression	↑	SP, SS, VIP not significant	↑GI motility	Kwon et al. (2021)
Yorkshire sows	Modified *Bazhen*	Normal control	n/a	n/a	1 week	↑NO, GAST	↑	↑ Piglet birth, milk yield	Lactating effect	Geng et al. (2021)
								↓Total labor course, farrowing interval		
SD rats	Chinese herb solid drink	Slow transit constipation	Lactulose	Intragastric	21–42 days	↑Fecal quality, the moisture content of feces, ITR	↑	↑GAST, SP	Laxative effect	Deng et al. (2021)
								↓VIP		
SD rats	*Zhishi-baizh*	Constipation	Loperamide	Oral	2 weeks	↑Fecal water content, fecal number	↑	↑SP, ATP, MLCK	Laxative effect	Yan et al. (2020)
SD rats	*Zuojin* pill	Chronic unpredictable mild stress model	Fluoxetine	Intragastric	5 weeks	↑ OFT	↑ (at high dose)	↑GAST	Antidepressant effect, ↑GI motility	Wang et al. (2020)
						↓ the sucrose preference		↓IL-1β, IL-6, TNF-α		
KM mice	*Guiren Runchang* granules	Slow transit constipation	Mosapride	Intragastric	2 weeks	↑ Stool weight, ITR	↑	↑C-kit	Laxative effect	Sun et al. (2020)
								↓AQP4		
KM mice	*Yangyin Tongmi* capsule with diphenoxylate	Constipation	n/a	Intragastric	2 weeks	↑ Stool number and moisture content, ITR	↑	↑SP, Ach	Laxative effect	Liu et al. (2020)
						↓ first black stool excretion time		↓GAST, SS, NO, AQP3, AQP8		
SD rats	*Buzhongyiqi* decoction	Constipation	Mosapride	Oral	5 d	↑ITR, number of stools, the epithelial surface of colon recovery	↑	↑GAST	Laxative effect	Ju et al. (2020)
								↓PGE_2_, IL-1,		
								COX-2, TNF-α		
SD rats	*Zhujie Hewei* granules	Reflux esophagitis	Omeprazole	Oral	4 weeks	↑Gastric pH	↓	↓GAST, VIP	Improvement in symptoms of reflux esophagitis	Qiu et al. (2019)
						↓Esophageal mucosal injury index score, inflammation score, macroscopic observation scores				
SD rats	*Chai-Qin-Cheng-Qi* decoction	Acute pancreatitis	Carbachol	Intragastric	30 h	↓Overall breakdown score, edema, inflammation, necrosis	↑	↓VIP, SP, iFABP	↑GI motility	Lin et al. (2016)
SD rats	*Yiqihuoxue* formula	Nonalcoholic fatty liver disease	The extracts mixed solution	Intragastric	5 weeks	↓TG, ALT	↑	↑GAST	Improved liver function, decreased fatty deposition in the liver	Chen et al. (2013)
Pigs (breed n/a)	Chinese medicine decoction	Heat-stressed model	n/a	Oral	6 d	↓Cor	↓	↑GCG ↓leptin, TSH-β, HAMP, GNRH1, IGF1, PTH, SS, SC, NPY	Relief of heat stress	Dong et al. (2012)
ICR mice	*Simotang*	Stress model	Mosapride	n/a	7 d	↑Gastric emptying, intestinal propulsion rate	↑	↓CCK-positive cells	↑GI motility	Cai et al. (2011)
SD rats	*Zuojin* pill, *Fanzuojin* pill, *Ganlusan*, *Zhuyu* pill	The gastric cold model	n/a	n/a	6 d	↓Injury index	↑	↑GAST	Improved gastric mucosal injury	Zhao et al. (2009)
Mongrel dogs	*DaiKenchuTou*	Normal control	n/a	Intraduodenum or jejunum	5–10 min after the end of phase III in the distal jejunum	↑Duodenum motility index, proximal jejunum motility index, distal jejunum motility index	Not signifi-cant		↑GI motility	Jin et al. (2001)

MDA: malonaldehyde; IL: interleukin; SYD: spleen yang deficiency; GI: gastrointestinal; GAST: gastrin; COX: cyclooxygenase; PGE_2_: prostaglandin E_2_; EGF: epidermal cell growth factor; OXA: orexin A; SOD: superoxide dismutase; GHRL: ghrelin; NPY: neuropeptide Y; GRP78: glucose-regulated protein 78; ER: endoplasmic reticulum; PERK: protein kinase R-like ER, kinase; elF2α: eukaryotic initiation factor2α; VIP: vasoactive intestinal peptide; SP: substance P; SS: somatostatin; ET: endothelin; SC: secretin; ITR: intestinal transit rate; NO: nitric oxide; TNF-α: tumor necrosis factor alpha; ATP: adenosine triphosphate; MLCK: myosin light chain kinase; OFT: open field test; AQP: aquaporin; STC: slow transit constipation; Ach: acetylcholine; iFABP: intestinal fatty acid binding protein; TG: triglyceride; ALT: alanine aminotransferase; Cor: cortisol; GCG: glucagon; TSH: thyrotropin; HAMP: antimicrobial peptide hepcidin; GNRH: gonadotropin-releasing hormone associated peptide; IGF: insulin-like growth factor; PTH: parathyroid hormone; CCK: cholecystokinin; KM: kunming; SD: Sprague-Dawley; n/a: not applicable; ↑: significant increase; ↓: significant decrease.

**TABLE 6:** Information on herbal medicines used in the included *in vivo* studies.

Herbal medicine	Extraction	Ingredients	Daily dose	Study ID
*Fuzi Lizhong* pill	Not extracted (Crude powder mixed with honey)	*Aconitum carmichaelii Debeaux [Ranunculaceae; Aconitum carmichaelii processed daughter root], Codonopsis pilosula (Franch.) Nannf. [Campanulaceae; Codonopsis pilosula dried root], Atractylodes lancea (Thunb.) DC. [Asteraceae; Atractylodes lancea dried rhizome], Zingiber officinale Roscoe [Zingiberaceae; Zingiber officinale dried rhizome], Glycyrrhiza uralensis Fisch. ex DC. [Fabaceae; Glycyrrhiza uralensis dried root and rhizome]. All botanical drugs are ground into fine powders at a ratio of 1: 2: 1.5: 1: 1*	50 mg of crude drug/mL (low dose), 150 mg of crude drug/mL (high dose), unclear daily amount	Zhang et al. (2021)
Invigorating qi and hemostasis formula	n/a	*Astragalus mongholicus Bunge [Fabaceae; Astragalus mongholicus dried root], Panax notoginseng (Burkill) F.H.Chen [Araliaceae; Panax notoginseng dried root], cuttlefish bone, Bletilla striata (Thunb.) Rchb.f. [Orchidaceae; Bletilla striata dried tuber], Rheum palmatum L. [Polygonaceae; Rheum palmatum dried root and rhizome]*	8.32 mg/kg, twice a day	Zhang et al. (2021)
*Ciji-Hua’ai-Baosheng* II	Water	*Salvia miltiorrhiza Bunge [Lamiaceae; Salvia miltiorrhiza] 50 g, Codonopsis pilosula (Franch.) Nannf. [Campanulaceae; Codonopsis pilosula dried root] 10 g, Poria cocos (Schw.) Wolf [Polyporaceae; Poria cocos sclerotium] 30 g, Citrus × aurantium L. [Rutaceae; Citrus × aurantium dried, immature fruit] 10 g, Hordeum vulgare L. [Poaceae; Hordeum vulgare dried germinated ripe fruit] 20 g, Ziziphus jujuba Mill. [Rhamnaceae; Ziziphus jujube dried ripe fruit] 25 g, Magallana gigas (Thunberg, 1793) 20 g, Fritillaria meleagris L. [Liliaceae; Fritillaria meleagris bulbus] 30 g*	3.25 g/mL (high dose),	Xi et al. (2021)
			1.625 g/mL (medium dose),	
			0.8125 g/mL (low dose), once a day	
*Dashanzha* pill	Not extracted (Mixed with 120 g of sucrose and 20 g of honey)	*Crataegus pinnatifida Bunge [Rosaceae; Crataegus pinnatifida dried ripe fruit] 200 g, fried Hordeum vulgare L. [Poaceae; Hordeum vulgare dried germinated ripe fruit] Triticum aestivum L. [Poaceae; Triticum aestivum outer fraction of the cereal grain, comprising the pericarp, seed coat (testa), nucellar tissue, and aleurone layer] 30 g*	0.25 mg/mL, twice a day	Liu et al. (2021)
*Tiantian* capsule	OTC	*n/a*	36 mg/kg (low dose),	Li et al. (2021)
			72 mg/kg (high dose), twice a day	
*Bojungikki Tang*	OTC	*Astragalus mongholicus Bunge [Fabaceae; Astragalus mongholicus dried root] 0.41 g, Panax ginseng C.A.Mey. [Araliaceae; Panax ginseng dried root] 0.30 g, Atractylodes lancea (Thunb.) DC. [Asteraceae; Atractylodes lancea dried rhizome] 0.46 g, Glycyrrhiza glabra L. [Fabaceae; Glycyrrhiza glabra Pharmaceutical] 0.34 g, Angelica gigas Nakai [Apiaceae; Angelica gigas root] 0.23 g, Citrus × aurantium L. [Rutaceae; Citrus × aurantium dried, immature fruit] 0.20 g, Actaea racemosa L. [Ranunculaceae; Actaea racemose dried rhizome and roots; harvested in the summer] 0.06 g, Bupleurum chinense DC. [Apiaceae; Bupleurum chinense dried root] 0.06 g*	n/a	Kwon et al. (2021)
Modified *Bazhen*	OTC	*15% of Astragalus mongholicus Bunge [Fabaceae; Astragalus mongholicus dried root], 15% of Atractylodes lancea (Thunb.) DC. [Asteraceae; Atractylodes lancea dried rhizome], 15% of Poria cocos (Schw.) Wolf [Polyporaceae; Poria cocos sclerotium], 11.25% of Glycyrrhiza uralensis Fisch. ex DC. [Fabaceae; Glycyrrhiza uralensis dried root and rhizome], 11.25% of Paeonia lactiflora Pall. [Paeoniaceae; Paeonia lactiflora dried root] 10% of Angelica sinensis (Oliv.) Diels [Apiaceae; Angelica sinensis dried root], 10% of Rehmannia glutinosa (Gaertn.) DC. [Orobanchaceae; Rehmannia glutinosa processed dried root tuber], 7.5% of Ziziphus jujuba Mill. [Rhamnaceae; Ziziphus jujube Pharmaceutical], 5% of Conioselinum anthriscoides ‘Chuanxiong’ [Apiaceae; Conioselinum anthriscoides dried rhizome]*	10 g, twice a day	Geng et al. (2021)
Chinese Herb Solid Drink	Water	*Plantago ovata Forssk. [Plantaginaceae; Plantago ovata cleaned, dried, ripe seed] 3 g, Cannabis sativa L. [Cannabaceae; Cannabis sativa dried ripe fruit] 2 g, Prunus amygdalus Batsch [Rosaceae; Prunus amygdalus refined fixed oil obtained by expression from the kernels] 1 g, Sesamum indicum L. [Pedaliaceae; Sesamum indicum dried ripe seed] 2 g, Resistant dextrin 1 g*	20 mg/mL, 3 times a day	Deng et al. (2021)
Zhishi-baizh	Water	*Citrus × aurantium L. [Rutaceae; Citrus × aurantium dried, immature fruit] 2 kg, Atractylodes macrocephala Koidz. [Asteraceae; Atractylodes macrocephala dried rhizome] 1 kg*	81 mg/kg, twice a day	Yan et al. (2020)
*Zuojin* pill	OTC	*n/a*	0.6 g/kg/d (low dose), 1.2 g/kg/d (high dose)	Wang et al. (2020)
*Guiren Runchang* granules	Water	*Anethum graveolens L. [Apiaceae; Anethum graveolens oil, seed oil] 10 g, Atractylodes lancea (Thunb.) DC. [Asteraceae; Atractylodes lancea dried rhizome] 25 g, Prunus persica (L.) Batsch [Rosaceae; Prunus persica dried ripe seed] 15 g, Cistanche deserticola Ma [Orobanchaceae; Cistanche deserticola dried fleshy stem with scales] 15 g, Citrus × aurantium L. [Rutaceae; Citrus × aurantium dried, immature fruit] 25 g, Magnolia officinalis Rehder and E.H.Wilson [Magnoliaceae; Magnolia officinalis dried stem bark, root bark or branch bark] 10 g, Typha angustifolia L. [Typhaceae; Typha angustifolia dried pollen] 15 g, Trogopterus xanthipes (Milne-Edwards, 1867)* *feces 12 g, Trichosanthes kirilowii Maxim. [Cucurbitaceae; Trichosanthes kirilowii processing product obtained from the seed] 20 g, Glycyrrhiza glabra L. [Fabaceae; Glycyrrhiza glabra dried root and rhizome] 6 g*	4.72 g/kg/d (low dose),	Sun et al. (2020)
			9.44 g/kg/d (middle dose),	
			18.88 g/kg/d (high dose)	
*Yangyin Tongmi* capsule with diphenoxylate	OTC	*n/a*	0.6 g/kg (low dose),	Liu et al. (2020)
			1.2 g/kg (high dose), once a day	
*Buzhongyiqi* decoction	Water	*Astragalus mongholicus Bunge [Fabaceae; Astragalus mongholicus dried root] 18g, Glycyrrhiza uralensis Fisch. ex DC. [Fabaceae; Glycyrrhiza uralensis dried root and rhizome] 9 g, Codonopsis pilosula (Franch.) Nannf. [Campanulaceae; Codonopsis pilosula dried root] 9g, Angelica sinensis (Oliv.) Diels [Apiaceae; Angelica sinensis dried root] 3 g, Citrus × aurantium L. [Rutaceae; Citrus × aurantium dried, immature fruit] 3 g, Actaea heracleifolia (Kom.) J.Compton [Ranunculaceae; Actaea heracleifolia dried rhizome] 6 g, Bupleurum falcatum L. [Apiaceae; Bupleurum falcatum root] 6 g, Atractylodes macrocephala Koidz. [Asteraceae; Atractylodes macrocephala dried rhizome] 9 g*	1.73 g/kg, twice a day	Ju et al. (2020)
*Zhujie Hewei* granules	Water and then concentrated to 12.5 g per bag	*Atractylodes macrocephala Koidz. [Asteraceae; Atractylodes macrocephala dried rhizome] 4.84 g, Rhaphiolepis bibas (Lour.) Galasso and Banfi [Rosaceae; Rhaphiolepis bibas dried leaf] 3.63 g, Gardenia jasminoides J.Ellis [Rubiaceae; Gardenia jasminoides Other] 3.63 g, Platycodon grandiflorus (Jacq.) A.DC. [Campanulaceae; Platycodon grandifloras dried root] 0.40 g*	1.3 g/kg (low dose), 2.6 g/kg (middle dose), 5.2 g/kg (high dose), once a day	Qiu et al. (2019)
*Chai-Qin Cheng-Qi* decoction	Not extracted (Lyophilized powder, 2 g/mL)	*Bupleurum falcatum L. [Apiaceae; Bupleurum falcatum root] 15 g, Scutellaria baicalensis Georgi [Lamiaceae; Scutellaria baicalensis dried root] 15 g, Rheum palmatum L. [Polygonaceae; Rheum palmatum dried root and rhizome] 20 g, Natrii Sulfas (mirabilite) 20 g, Magnolia officinalis Rehder and E.H.Wilson [Magnoliaceae; Magnolia officinalis dried stem bark, root bark or branch bark] 15 g, Citrus × aurantium L. [Rutaceae; Citrus × aurantium dried, immature fruit] 15 g, Bassia scoparia (L.) A.J.Scott [Amaranthaceae; Bassia scoparia dried ripe fruit] 15 g, Gardenia jasminoides J.Ellis [Rubiaceae; Gardenia jasminoides dried ripe fruit] 20 g*	20 g/kg, 2 h, 3 doses a day	Lin et al. (2016)
*Yiqihuoxue* formula	Water	*Gardenia jasminoides J.Ellis [Rubiaceae; Gardenia jasminoides dried ripe fruit], Rhodiola rosea L. [Crassulaceae; Rhodiola rosea dried roots and rhizomes], Curcuma longa L. [Zingiberaceae; Curcuma longa dried root tuber], Ligustrum lucidum W.T.Aiton [Oleaceae; Ligustrum lucidum dried ripe fruit]. The dose ratio was of 1∶1∶1∶1*	1 mL/100 g of body weight, every day	Chen et al. (2013)
Chinese medicine decoction	Water	*Phellodendron amurense Rupr. [Rutaceae; Phellodendron amurense dried bark], Atractylodes lancea (Thunb.) DC. [Asteraceae; Atractylodes lancea dried rhizome], Agastache rugosa (Fisch. and C.A.Mey.) Kuntze [Lamiaceae; Agastache rugose dried aerial part], Gypsum fibrosum. All were combined in a dry weight ratio of 1:1:1:0.5.*	0.15 g/kg/d	Dong et al. (2012)
*Simotang*	Water, 0.5 mg/mL	*Citrus × aurantium L. [Rutaceae; Citrus × aurantium dried, immature fruit], Dolomiaea costus (Falc.) Kasana and A.K.Pandey [Asteraceae; Dolomiaea costus dried root], Areca catechu L. [Arecaceae; Areca catechu dried pericarp]*	1.2 g/kg	Cai et al. (2011)
*Zuojin* pill	??	*Coptis chinensis Franch. [Ranunculaceae; Coptis chinensis; dried rhizome]: Tetradium ruticarpum (A.Juss.) T.G.Hartley [Rutaceae; Tetradium ruticarpum dried and nearly ripe fruit] = 6:1 per gram*	2 g, once a day	Zhao et al. (2009)
*Fanzuojin* pill		*Coptis chinensis Franch. [Ranunculaceae; Coptis chinensis; dried rhizome]: Tetradium ruticarpum (A.Juss.) T.G.Hartley [Rutaceae; Tetradium ruticarpum dried and nearly ripe fruit] = 1:6, per gram*	2.69 g, once a day	
*Ganlu* powder		*Coptis chinensis Franch. [Ranunculaceae; Coptis chinensis; dried rhizome]: Tetradium ruticarpum (A.Juss.) T.G.Hartley [Rutaceae; Tetradium ruticarpum dried and nearly ripe fruit] = 2:1, per gram*	2.99 g, once a day	
*Zhuyu* pill		*Coptis chinensis Franch. [Ranunculaceae; Coptis chinensis; dried rhizome]: Tetradium ruticarpum (A.Juss.) T.G.Hartley [Rutaceae; Tetradium ruticarpum dried and nearly ripe fruit] = 1:1, per gram*	2.93 g, once a day	
*DaiKenchuTou*	OTC	*n/a*	0.5, 1.5, or 3.0 g, unclear daily amount	Jin et al. (2001)

OTC, over-the-counter medicine; n/a, not applicable.

### 3.3 Assessment of risk of bias

**FIGURE 3:**
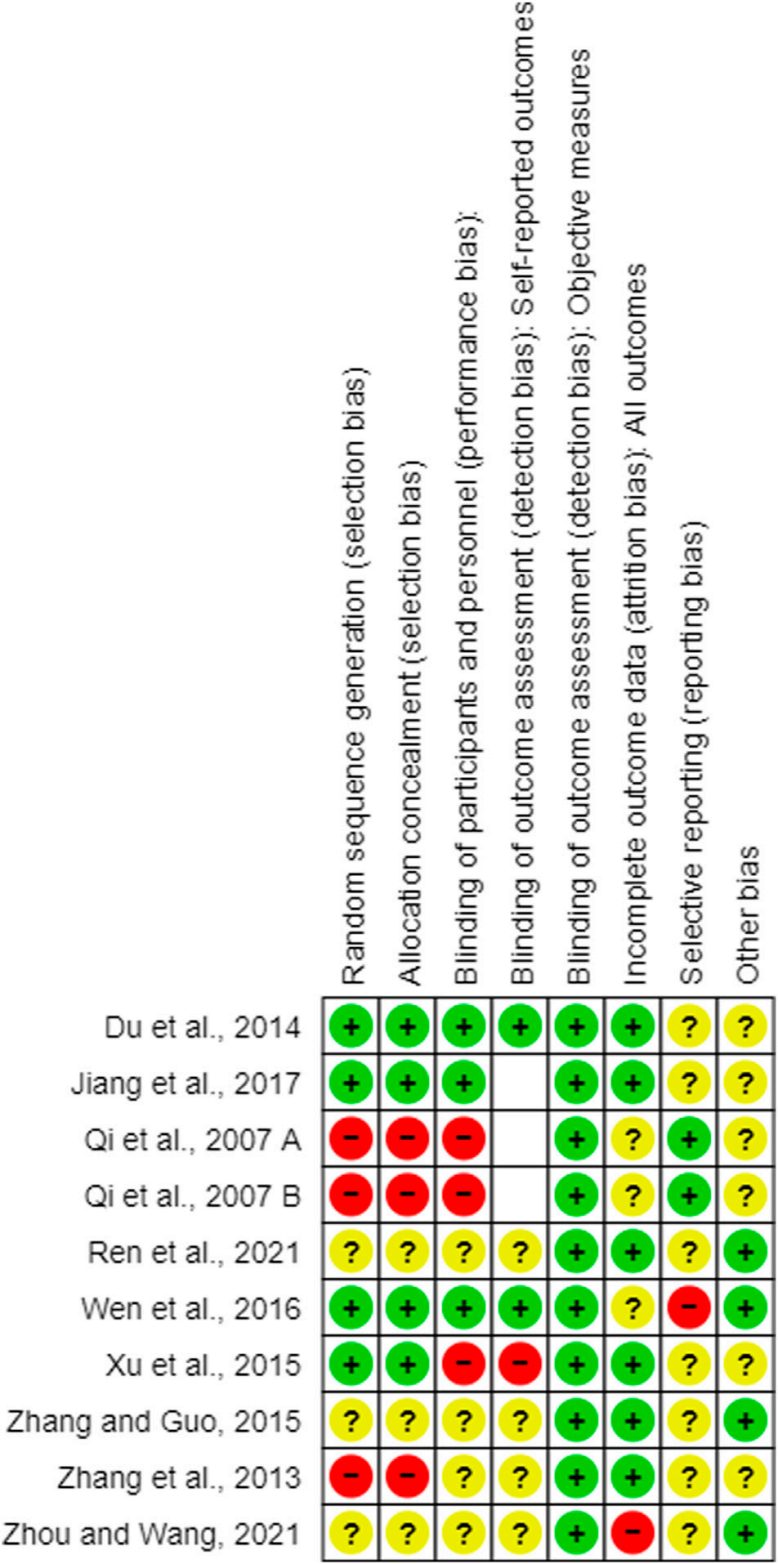
Summary of the risk of bias. +: low risk of bias; ?: unclear risk of bias; -: high risk of bias, empty slot: not applicable.

**FIGURE 4:**
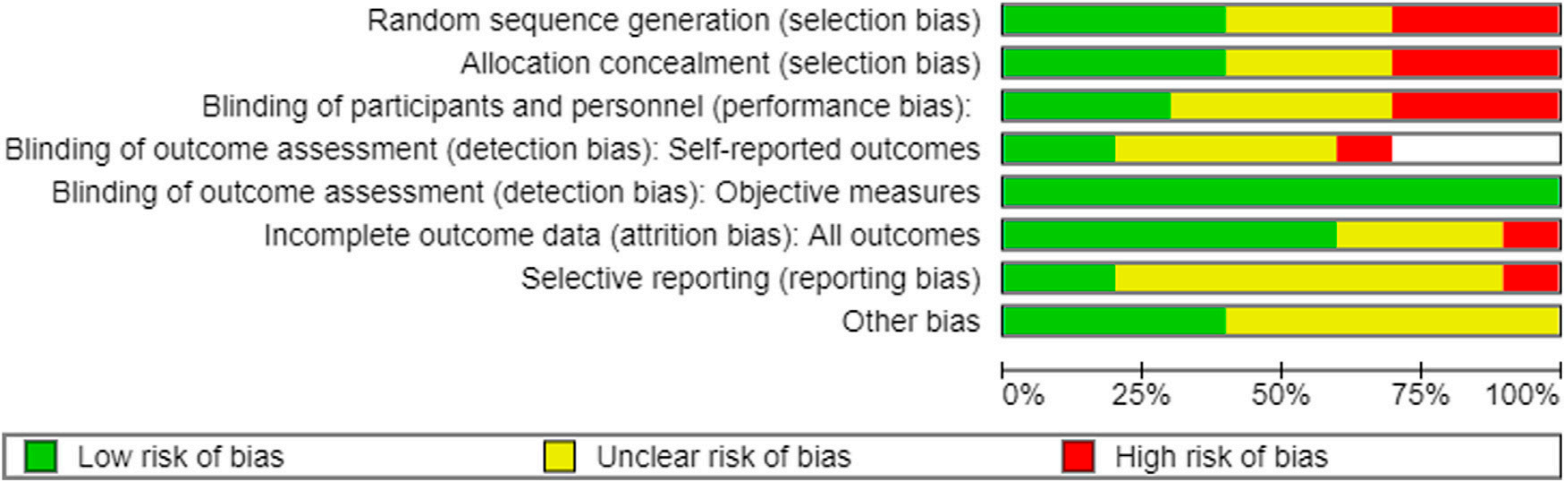
Graph of the risk of bias. Empty slot: not applicable.

All RCTs included in this review were assessed for risk of bias using the Cochrane risk of bias tool. The results of the risk of bias assessment are shown in Figures 3,4. The quality of animal studies was assessed using ARRIVE checklist and provided as Supplementary Material S3.

#### 3.3.1 Random sequence generation

Four of the 10 studies (Du et al., 2014; Xu et al., 2015; Wen et al., 2016; Jiang et al., 2017) used the random sequence generation method. Three studies (Qi et al., 2007; Qi et al., 2007; Zhang et al., 2013) had a high level of selection bias. Qi et al. (2007A, B) generated the sequence according to the patients’ admission time, and Zhang et al. (2013) generated the sequence using the visiting order. The other three studies did not report the random sequencing method used (Zhang and Guo, 2015; Ren et al., 2021; Zhou and Wang, 2021).

#### 3.3.2 Allocation

Four studies (Du et al., 2014; Xu et al., 2015; Wen et al., 2016; Jiang et al., 2017) showed a low risk of selection bias using a sealed envelope (Du et al., 2014; Jiang et al., 2017), PEMS 3.1 software (Wen et al., 2016), or a random number table (Xu et al., 2015) as the allocation method. Three studies had a high risk of bias using an open random allocation schedule (Qi et al., 2007 A; Qi et al., 2007 B) or the patients’ visiting order (Zhang et al., 2013) as allocation methods. Three studies (Zhang and Guo, 2015; Ren et al., 2021; Zhou and Wang, 2021) did not provide sufficient information to judge allocation bias.

#### 3.3.3 Blinding of participants and personnel

Three studies (Du et al., 2014; Wen et al., 2016; Jiang et al., 2017) performed clinical experiments in a double-blind manner. Three studies (Qi et al., 2007A; Qi et al., 2007 B; Xu et al., 2015) used placebos that patients could easily recognize as a control drug (normal saline in one study (Xu et al., 2015) and a normal solution in two studies [Qi et al., 2007A; Qi et al., 2007B]); therefore, they were judged to have a high risk of performance bias. Finally, four studies (Zhang et al., 2013; Zhang and Guo, 2015; Ren et al., 2021; Zhou and Wang, 2021) did not provide sufficient information to judge bias.

#### 3.3.4 Blinding of outcome assessment

##### 3.3.4.1 Blinding of outcome assessment of self-reported measures

Three studies (Jiang et al., 2017; Qi et al., 2007 A; Qi et al., 2007 B) did not use subjective self-reported outcomes. Moreover, two studies (Du et al., 2014; Wen et al., 2016) were double-blinded, and the patient-reported outcomes had a low risk of bias, as the patients were unaware of their treatment allocation. In one study (Xu et al., 2015), the patients could not be blinded to the allocation; therefore, self-reported measures were used, which have a high risk of bias. The other four studies (Zhang et al., 2013; Zhang and Guo, 2015; Ren et al., 2021; Zhou and Wang, 2021) did not have sufficient information to confirm whether self-reported outcomes were assessed in a blinded manner.

##### 3.3.4.2 Blinding of the outcome assessment of objective measures

All included RCTs used objective biochemical findings that were automatically recorded without intervention by the assessors; therefore, we judged them as having a low risk of detection bias.

#### 3.3.5 Attrition bias

The difference in the number of dropouts between the treatment and control groups in one study (Zhou and Wang, 2021) was statistically significant; therefore, we judged it to have a high risk of attrition bias. Three studies (Qi et al., 2007A; Qi et al., 2007B; Wen et al., 2016) did not report patient dropouts. The other six studies (Zhang et al., 2013; Du et al., 2014; Xu et al., 2015; Zhang and Guo, 2015; Jiang et al., 2017; Ren et al., 2021) reported that the difference in the number of patients who dropped out between the treatment and control groups was not statistically significant (low risk of bias).

#### 3.3.6 Selective bias

Two studies (Qi et al., 2007A; Qi et al., 2007B) reported the existence of an experimental protocol; therefore, they had a low risk of bias. Furthermore, in one study (Wen et al., 2016), one variable (bowel sounds) in the Methods section was not described in the Results section; therefore, it was judged as a high risk. The other seven studies (Zhang et al., 2013; Du et al., 2014; Xu et al., 2015; Zhang and Guo, 2015; Jiang et al., 2017; Ren et al., 2021; Zhang et al., 2021) were judged to have an unclear risk of bias due to insufficient reporting of the outcomes.

#### 3.3.7 Others

Four studies (Zhang and Guo, 2015; Wen et al., 2016; Ren et al., 2021; Zhou and Wang, 2021) reported that they were free of conflicts of interest, whereas the other six studies (Qi et al., 2007; Qi et al., 2007; Zhang et al., 2013; Du et al., 2014; Xu et al., 2015; Jiang et al., 2017) did not report any conflicts of interest.

### 3.4 FP growth algorithm

**FIGURE 5:**
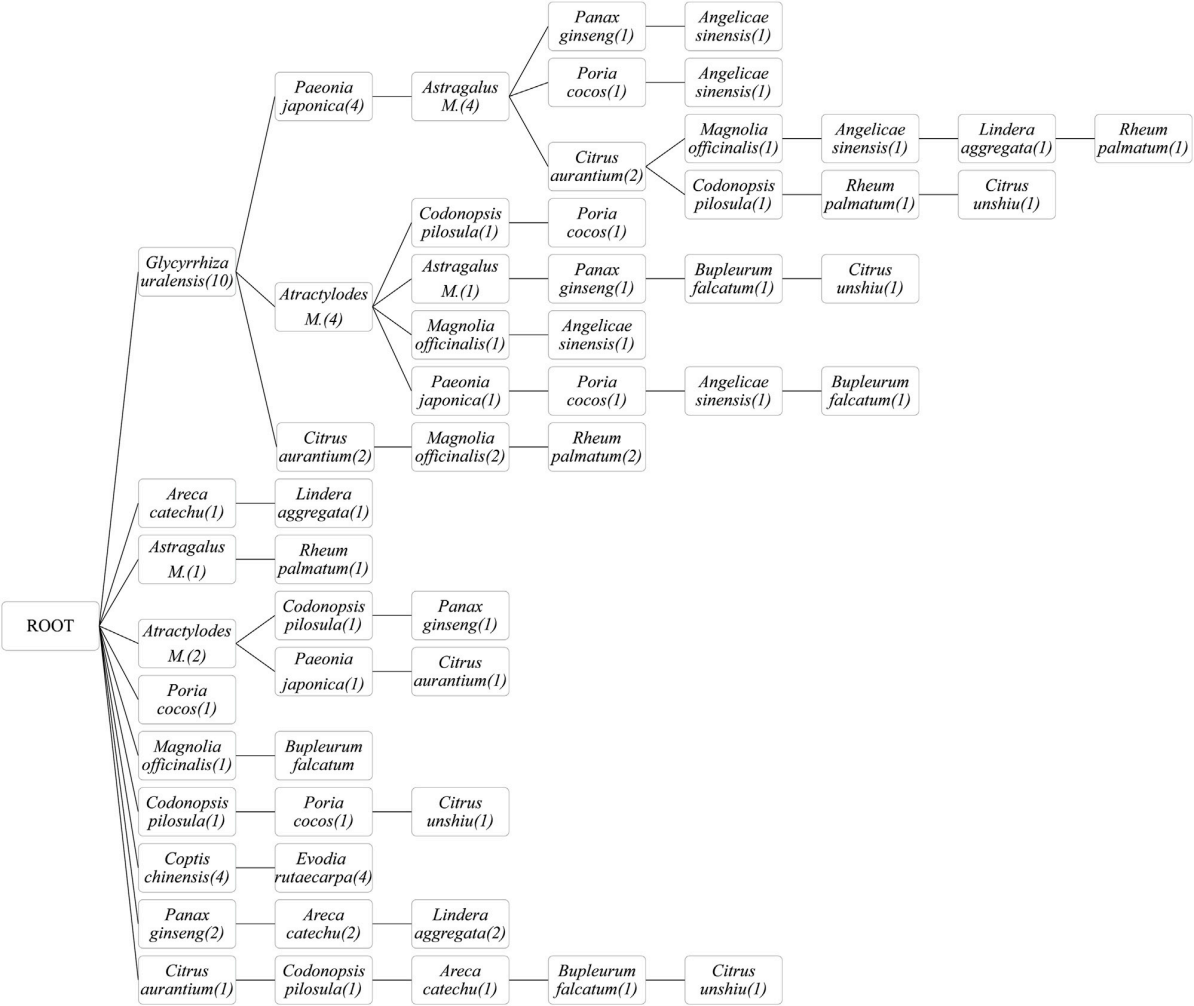
Frequent pattern growth algorithm of herbal medicines used. *Astragalus M.*: *Astragalus membranaceus*; *Atractylodes M.*: *Atractylodes macrocephala*.

The ingredients of the herbal medicines were extracted, mined, and analyzed, and the FP growth algorithm identified the most frequently used botanical drugs and their combinations (Figure 5). Among the included studies, *Glycyrrhiza uralensis* was most frequently used, followed by *Paeonia japonica*, *Atractylodes macrocephala*, *Citrus aurantium,* and *Astragalus membranaceus*. Combinations of three botanical drugs (*Glycyrrhiza uralensis*, *Paeonia japonica,* and *Astragalus membranaceus*), two botanical drugs (*Glycyrrhiza uralensis* and *Atractylodes macrocephala*), and two other botanical drugs (*Glycyrrhiza uralensis* and *Angelicae sinensis*) were most frequently used.

## 4 Discussion

**FIGURE 6:**
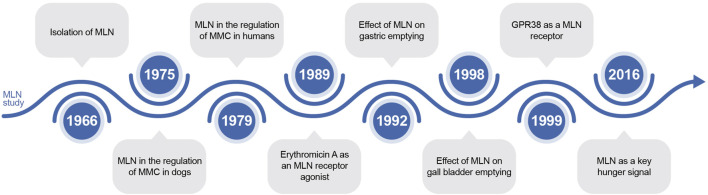
The timeline of discoveries related to motilin. MLN: motilin; GPR 38: G protein-coupled receptor 38.

MLN was first identified due to its prokinetic effects. In 1966, Brown et al. found that duodenal alkalinization increased the motility of denervated gastric pouches (Brown et al., 1966). In 1975, MLN was found to regulate the migrating motor complex in dogs, and this function was identified in 1979 in humans (Code and Marlett, 1975; Vantrappen et al., 1979). Furthermore, in 1989, the antibiotic ER was discovered as an MLN receptor agonist (Peeters et al., 1989). Subsequently, the receptor for MLN, the G protein-coupled receptor 38, was discovered in 1999 (Feighner et al., 1999). Notably, the scope of MLN studies has expanded to the potential involvement of MLN in signaling hunger in 2016 (Tack et al., 2016). A summary of the history of MLN is reported in Figure 6.

MLN can influence the functioning of various parts of the body, including the GI tract, gallbladder, pancreas, rectum, and brain (Depoortere et al., 1997; Thielemans et al., 2001; Luiking et al., 2002; Kamerling et al., 2003; Sanger et al., 2011). The distribution of MLN and its receptors varies in mammals. In humans, MLN receptors are widespread. Moreover, although MLN is most abundantly expressed in the human gastroduodenal region, their actions might be related to the part of brain function (Depoortere et al., 1997; Thielemans et al., 2001; Sanger et al., 2013; Zhang et al., 2023). Although MLN is widely distributed in mammals, it is not active in all species. For example, in most rodents, due to genetic variations in the genes encoding MLN or its receptors during evolution, the gene structures have changed, rendering them functionally inactive (Sanger, 2022). There are studies that have measured MLN in rodent models that have developed specific genetic mutations (Delinsky et al., 2004; Sanger, 2022), and there is a report that the ghrelin receptor responds to extremely high concentration of MLN leading to improvement of gastrointestinal motility in rodents (Kim and Kim, 2019). The secretion and activity of motilin in rodent are still controversial.

### 4.1 Main findings

In most of the studies in this review (23 of 29), MLN levels were increased by herbal medicines. Furthermore, when serum MLN levels increase due to herbal medicines, the human body reacts by accelerating GI movement. For example, duodenal and jejunal motility increased, and indigestion and constipation were alleviated.

The effects of herbal medicines in animal studies were diverse, extending beyond their therapeutic impacts on GI motility and digestive function. For instance, platelet activation was reduced when MLN levels increased; an inhibitory effect on cancer was demonstrated in a chemotherapy model using Kunming mice; endoplasmic reticulum stress was reduced; and depression alleviated.

Notably, the change in serum MLN was statistically significant in all studies except for 1 study (Jin et al., 2001), and herbal medicine acted on the GI tract. Furthermore, the effects on the digestive system were consistent with the function of MLN, which increases GI motility, suggesting that MLN might be a powerful mediator of the actions of herbal medicine on GI motility. These data may provide evidence for the effect of herbal medicines on digestive system dysfunction.

### 4.2 Correlation between biochemical findings and MLN levels

In the present review, laboratory data, including serum levels of endogenous hormones, enzymes, neurotransmitters, inflammatory factors, gene expression markers, nutrient factors, and carrier proteins, may be correlated with changes in serum MLN levels. As shown in Tables 3 and 5, the biochemical data showed positive and negative trend, or no correlation with changes in plasma MLN levels. The relationship between various biochemical substances and plasma MLN levels are summarized in Table 7.

**TABLE 7:** Summary of the correlation between plasma motilin levels and biochemical findings.

Item	Origin of data	Positive correlation	Negative correlation	No correlation
Endogenous hormones	Human	Ghrelin	Corticotropin-releasing hormone	Gastrin, vasoactive intestinal peptide, somatostatin
	Animal	Ghrelin, epidermal growth factor, leptin, gonadotropin-releasing hormone associated peptide 1, insulin-like growth factor, parathyroid hormone, secretin	glucagon, cortisol	Gastrin, vasoactive intestinal peptide, somatostatin
Enzymes	Animal	Superoxide dismutase	Protein kinase R-like endoplasmic reticulum kinase, cyclooxygenase 2, alanine aminotransferase	n/a
Gene expression markers	Animal	C-kit expression, antimicrobial peptide hepcidin	Eukaryotic initiation factor 2α	n/a
Inflammation factors	Human	n/a	Serum inflammatory factor reactive protein, IL-6	n/a
	Animal	Thyrotropin beta, malonaldehyde, IL-1α	IL-1β, IL-6, tumor necrosis factor alpha	n/a
Immunoglobulins	Human	IgG, IgM, IgA	n/a	n/a
Neurotransmitters	Animal	Acetylcholine, orexin A, substance P	Endothelin-1, neuropeptide Y	Nitric oxide
Nutrient factors	Animal	n/a	Glucose-regulated protein78, triglyceride, prostaglandin E_2_	n/a
Carrier proteins	Animal	n/a	AQP3, AQP4, APQ8, intestinal fatty acid binding protein	n/a
Other factors	Animal	Piglet births, milk yield	Total labor course, farrowing interval, platelet aggregation rate	n/a

n/a: not applicable; IL: interleukin; IG: immunoglobulin; AQP: aquaporin.

The correlation between certain biochemical findings and MLN levels in the present study was not in accordance with the findings of previous studies. For example, somatostatin was reported to be negatively correlated with MLN levels in studies by Deloose et al. (2019) and Kitazawa and Kaiya (2021), whereas the present review showed no such correlation. Furthermore, here, ghrelin showed a positive correlation with MLN levels; however, Kitazawa and Kaiya (2021) reported a negative correlation and Deloose et al. (2019) reported no correlation. Other biochemical findings, such as acetylcholine, triglyceride, prostaglandin E_2,_ and secretin levels, also showed different correlations among the three studies (the present review, Deloose et al., 2019; Kitazawa and Kaiya, 2021). The comparison of correlations between the biochemical findings and MLN levels in 3 reviews (Deloose et al., 2019; Kitazawa and Kaiya, 2021; current review) are shown in Table 8.

**TABLE 8:** Comparison of changes in serum motilin levels in three reviews.

Items	Deloose et al. (2019)	Kitazawa and Kaiya (2021)	Current review
Gastrin	n/c	n/c	n/c
Somatostatin	Negative	Negative	n/c
Ghrelin	n/c	Negative	Positive
Acetylcholine	No report	Positive	Positive
Secretin	Negative	n/c	Positive
Triglyceride	Positive	n/c	Negative
Prostaglandin E_2_	No report	Positive	Negative

n/c: no correlation.

The different results of biomarkers affecting serum MLN levels may be attributed to variations in studies conducted using different animals. MLN and its receptor have undergone variation across species during evolution, and the relationships between biomarkers and serum MLN levels in various animals are not fully understood (Kitazawa and Kaiya, 2021). Notably, previous studies have reported that secretin does not affect serum MLN levels in dogs (Lee et al., 1980; Poitras et al., 1993). In this review and in Deloose et al. (2019), however, secretin showed a negative correlation with serum MLN levels, and this result has been replicated in a study using pigs and in human clinical studies (Mitznegg et al., 1977; Jenssen et al., 1986; Dong et al., 2012).

Among macronutrients, lipids, such as triglycerides, are known to have contradictory effects on GI motility. Miedzybrodzka et al. (2021) reported that the long-chain fatty acid receptor FFA1 and the monoacylglycerol receptor GPR119 stimulated MLN secretion. However, a chronic high-fat diet increases glucose-dependent insulinotropic polypeptide (GIP) and glucagon-like peptide-1 (GLP-1) secretion (Wang et al., 2015). Notably, GIP and GLP-1 decrease GI motility (Thor et al., 1987; Hellström et al., 2008).

### 4.3 Botanical drugs and their combinations according to the FP growth algorithm

We analyzed the most commonly used botanical drugs and their combinations according to the FP growth algorithm and found that *Glycyrrhiza uralensis*, *Paeonia japonica*, *Atractylodes macrocephala*, *Citrus aurantium,* and *Astragalus membranaceus* were most frequently used. When analyzing frequently combined botanical drugs, the combinations of *Glycyrrhiza uralensis* with *Paeonia japonica* and *Astragalus membranaceus*, *Glycyrrhiza uralensis* with *Atractylodes macrocephala*, and *Glycyrrhiza uralensis* with *Angelicae sinensis* were most common. Furthermore, *Glycyrrhiza uralensis*, also known as licorice, is the most widely used botanical drug that harmonizes with the characteristics of other botanical drugs in traditional Chinese medicine (Jiang et al., 2020). A traditional Chinese quote states that “nine out of ten formulas contain licorice.” Licorice is usually combined with other botanical drugs owing to its balancing effect (Wang and Su, 2002). Moreover, *Paeonia japonica* has been shown to exert prokinetic effects by increasing gastric emptying and intestinal transit due to increased MLN levels in a rat model (Mu et al., 2020). Further, *Paeonia japonica* and *Glycyrrhiza uralensis* have been shown to inhibit the pacemaker potential of interstitial cells of Cajal, regulating GI motility, and this is associated with MLN (Choi et al., 2022). *Atractylodes macrocephala* increases the levels of MLN, resulting in improved gastric emptying with the activation of the vagal pathway (Zhang et al., 2021). Furthermore, the main active ingredients of *Citrus aurantium* (hesperidin or *Fructus aurantii*) promotes GI movement and gastric motility by regulating the secretion of MLN in a rat model of functional dyspepsia (Zhu et al., 2020; Jia et al., 2022). Finally, Yan et al. showed elevated MLN levels in patients with gastric cancer (Yan et al., 2009), and *Astragalus membranaceus* has been shown to reduce MLN levels and inactivate the NF-κB signaling pathway, indicating its protective effect on chronic atrophic gastritis (Tang et al., 2022).

### 4.4 Limitations

This study has several limitations. First, the studies included in this review were mostly conducted in China, which might have caused publication bias. Second, most *in vivo* studies were performed using mice and rats. Because of the pseudogenization of MLN genes in rodents, it is difficult to directly apply the results of *in vivo* studies to humans or other mammals in which MLN and its receptors have retained their function. Third, the heterogeneity between herbal medicines was high, which may be one of the reasons why a meta-analysis has not been conducted. Fourth, detailed information such as effective chemical profiles and quality control measures for defining the composition of the study material in original studies were lacking. Fifth, herbal medicines in this review were administered with the secondary variable for altering serum motilin, so there was lack of direct evidence for their causal relationship between the effect of herbal medicine and MLN. Finally, although we have described the herbal medicines in validated taxonomical way, we acknowledge that our efforts may not fully meet the requirements outlined in the ConPhyMP statement. In future studies, we are committed to strengthening our efforts to characterize the profile of herbal medicines used in clinical and animal studies investigations in line with the importance highlighted in the ConPhyMP statement to express our findings more comprehensively.

### 4.5 Strengths and future perspectives

To the best of our knowledge, this is the first systematic review investigating the effect of herbal medicine and its influence on MLN. We extensively reviewed various herbal medicines and diseases that can be affected by MLN. We also discussed how these herbal medicines may affect hormonal changes, thereby contributing to the scientific understanding of herbal medicine and facilitating its transition to evidence-based applications. Additionally, we used an FP growth pattern algorithm to identify the most used combinations of botanical drugs for managing plasma MLN levels.

In the future, more in-depth research is needed to determine the mechanism by which composition of herbal medicine and its metabolites affect MLN. For example, herbal medicine might be related to the action of MLN receptor. Furthermore, for clinical applications, MLN studies are needed in mammals without the pseudogenization of MLN genes, including humans. In addition, further research is needed to investigate the mutual relationships between the constituent botanical drugs.

Considering the antidepressant effects of herbal medicines, MLN is thought to be involved in serotonin release. Notably, through both vagus efferent neurons and serotonin pathways, MLN can facilitate GI tract movement (Takahashi, 2012). Thus, the in-depth mechanism responsible for these brain-gut connections requires further investigation. Furthermore, human ghrelin and MLN, the major gut hormones, act on structurally similar G-protein-coupled receptors and exhibit 50% overall identity with each other (Chen and Tsai, 2012; Sanger and Furness, 2016). Moreover, both hormones have functionally similar actions in initiating the migrating motor complex in the stomach, accelerating gastric emptying, and inducing “gastric hunger” (Chen and Tsai, 2012). Further studies analyzing the interrelationships between MLN and other gut hormones are required.

### 4.6 Conclusion

We found that most herbal medicines may be related to increase and decrease in serum MLN levels and bring various symptomatic improvement. Through the regulation of MLN, herbal medicines may exert a therapeutic effect on GI symptoms such as diarrhea, dyspepsia and gastroesophageal reflux disease, and various disorders including autonomic dysfunction and depression in human. Moreover, we found evidence of herbal medicines’ anti-cancer, anti-inflammatory, and anti-stress effects in animal models. This systematic review suggests that herbal medicine may be useful and beneficial in treating MLN-related disorders. Further studies are needed to investigate direct evidence of a therapeutically-relevant action of herbal medicine to MLN, and specify their metabolites in MLN regulation in animal models and humans.

## Data Availability

The original contributions presented in the study are included in the article/Supplementary Material, further inquiries can be directed to the corresponding author.
