# Supplementary material for: The influence of herbal medicine on serum motilin and its effect on human and animal model: a systematic review
Source: Front Pharmacol. 2023 Dec 14;14:1286333. doi: 10.3389/fphar.2023.1286333 (PMC10755953; doi:10.3389/fphar.2023.1286333)
Supplement: Supplementary file 1 [file Table1.DOCX]

**Supplementary material 2.** Specific information on motilin change

| Human studies | Concentration value of control group | Concentration value of treatment group | Concentration unit | p-value |
| --- | --- | --- | --- | --- |
| Zhou and Wang, 2021 | 370.45 ± 30.45 | 277.54 ± 27.45 | Mean ± SD, ng/mL | <0.001 |
| Ren et al., 2021 | 594.35 ± 36.17 | 282.34 ± 21.37 | N/R, μg/L | <0.001 |
| Jiang et al., 2017 | 51.37 ± 6.60 | 57.69 ± 9.03 | Mean ± SEM, pg/mL | <0.001 |
| Wen et al., 2016 | 727.48 ± 926.20 | 457.13 ± 824.15 | Mean ± SD, ng/L | <0.05 |
| Zhang and Guo, 2015 | 371.58 ± 25.45 | 90.49 ± 36.14 | Mean ± SD, mg/L | <0.01 |
| Xu et al., 2015 | 230 ± 60 | 322 ± 78 | Mean ± SD, ng/L | <0.05 |
| Du et al., 2014 | 87.67 ± 23.50 | 197.46 ± 26.37 | Mean ± SD, ng/L | <0.05 |
| Zhang et al., 2013 | 369.4 ± 31.6 | 499.8 ± 41.8 | Mean ± SD, mg/L | <0.05 |
| Qi et al., 2007* | 96 ± 30 | 143 ± 33 | Mean ± SD, ng/L | <0.001 |
| Animal studies | Concentration value of control group | Concentration value of treatment group | Concentration unit | p-value |
| Z. Zhang et al., 2021* | 1760 ± 210 | 1200 ± 180 | Mean ± SD, pg/mL | <0.01 |
| C. H. Zhang et al., 2021* | 120.1 ± 6.7 | 136.1 ± 9.2 | Mean ± SD, pg/mL | <0.05 |
| Xi et al., 2021* | 297.3 ± 12.3 | 363.1 ± 24.5 | Mean ± SD, pg/mL | <0.01 |
| Liu et al., 2021* | 42.0 ± 0.1 | 61.3 ± 0.7 | Mean ± SD, pg/mL | <0.01 |
| Li et al., 2021* | 594.2 ± 153.4 | 792.8 ± 244.4 | Mean ± SD, pg/mL | <0.01 |
| Kwon et al., 2021* | 1.0 | 1.2 ± 0.025 | Mean ± SD, N/R | <0.05 |
| Geng et al., 2021* | 282.4 ± 24.1 | 343.4 ± 60.6 | Mean ± SD, pg/mL | <0.05 |
| Deng et al., 2021* | 198.6 ± 18.8 | 414.7 ± 31.5 | Mean ± SD, pg/mL | <0.001 |
| Yan et al., 2020* | 42.3 ± 5.7 | 67.5 ± 7.0 | Mean ± SD, pg/mL | <0.01 |
| Wang et al., 2020 | 28.5 ± 2.0 | 37.0 ± 2.5 | Mean ± SD, pg/mL | <0.05 |
| Sun et al., 2020* | 12.4 ± 0.6 | 12.5 ± 1.2 | Mean ± SD, pg/mL | <0.01 |
| Liu et al., 2020* | 665 ± 332 | 1176 ± 508 | Mean ± SD, pg/mL | <0.01 |
| Ju et al., 2020* | 11 ± 1 | 20 ± 7 | Mean ± SD, pg/mL | <0.01 |
| Qiu et al., 2019 | 678.81 ± 92.92 | 467.94 ± 41.71 | Mean ± SD, ng/L | < 0.01 |
| Lin et al., 2016* | 206 ± 22 | 192 ± 6 | Mean ± SD, pg/mL | <0.01 |
| Chen et al., 2013 | 0.21 ± 0.02 | 0.28 ± 0.03 | Mean ± SD, ng/mL | <0.05 |
| Dong et al., 2012 | N/R | N/R | N/R | <0.05 |
| Cai et al., 2011* | 88 ± 8 | 136 ± 6 | Mean ± SD, pg/mL | <0.01 |
| Zhao et al., 2009 | 209.14 ± 15.73 | 229.82 ± 16.33 (a) | Mean ± SD, pg/mL | <0.01 |
|  | 209.14 ± 15.73 | 238.75 ± 21.54 (b) | Mean ± SD, pg/mL | <0.01 |
|  | 209.14 ± 15.73 | 231.68 ± 18.34 (c) | Mean ± SD, pg/mL | <0.01 |
|  | 209.14 ± 15.73 | 236.78 ± 22.56 (d) | Mean ± SD, pg/mL | <0.01 |
| Jin et al., 2001 | 230 | 260 | Mean, pg/mL | Insignificant |

SD: Standard deviation; N/R: Not reported; SEM: Standard error of the mean; (a): *Zuojin* pill group; (b): *Fanzuojin* pill group; (c): *Ganlusan* group; (d): *Zhuyu* pill group; *: Data was extracted from the chart.
