# Supplementary material for: The influence of herbal medicine on serum motilin and its effect on human and animal model: a systematic review
Source: Front Pharmacol. 2023 Dec 14;14:1286333. doi: 10.3389/fphar.2023.1286333 (PMC10755953; doi:10.3389/fphar.2023.1286333)
Supplement: Supplementary file 2 [file Table2.DOCX]

**Supplementary material 3.** ARRIVE checklist for animal studies

| Item |  | Zhang et al. 2021 | Zhang et al. 2021 | Xi et al. 2021 | Liu et al. 2021 | Li et al. 2021 | Kwon et al. 2020 | Geng et al. 2021 | Deng et al. 2021 | Yan et al. 2020 | Wang et al, 2020 | Sun et al. 2020 | Liu et al. 2020 | Ju et al. 2020 | Qiu et al. 2019 | Lin et al. 2016 | Chen et al. 2013 | Dong et al, 2012 | Cai et al, 2011 | Zhao et al, 2009 | Jin et al, 2001 |
| --- | --- | --- | --- | --- | --- | --- | --- | --- | --- | --- | --- | --- | --- | --- | --- | --- | --- | --- | --- | --- | --- |
| Study design | (A) | Y | Y | Y | Y | Y | Y | Y | Y | Y | Y | Y | Y | Y | Y | Y | Y | Y | Y | Y | Y |
|  | (B) | Y | Y | Y | Y | Y | Y | Y | Y | Y | Y | Y | Y | Y | Y | Y | Y | Y | Y | Y | Y |
| Sample size | (C) | Y | Y | Y | Y | Y | Y | Y | Y | Y | Y | Y | Y | Y | Y | Y | Y | Y | Y | Y | Y |
|  | (D) | N | N | N | N | N | N | N | N | N | N | N | N | N | N | N | N | N | N | N | N |
| Inclusion and exclusion criteria | (E) | Y | N | N | N | N | N | N | N | N | N | N | N | N | N | N | N | N | N | N | N |
|  | (F) | N | N | N | N | N | N | Y | N | N | N | N | N | Y | N | Y | N | N | N | N | N |
|  | (G) | Y | Y | Y | Y | Y | Y | Y | Y | Y | N | Y | Y | Y | Y | Y | Y | Y | Y | Y | Y |
| Randomization | (H) | N | N | N | N | N | N | N | N | N | N | N | N | N | N | N | N | Y | N | N | N |
|  | (I) | N | N | N | N | N | N | N | N | N | Y | N | N | N | N | N | N | Y | N | N | N |
| Blinding | (J) | N | N | N | N | N | N | Y | N | N | N | N | N | Y | N | Y | N | N | N | N | N |
| Outcome measures | (K) | Y | Y | Y | Y | Y | Y | Y | Y | Y | Y | Y | Y | Y | Y | Y | Y | Y | Y | Y | Y |
|  | (L) | Y | Y | Y | Y | Y | Y | Y | Y | Y | Y | Y | Y | Y | Y | Y | Y | Y | Y | Y | Y |
| Statistical methods | (M) | Y | Y | Y | Y | Y | Y | Y | Y | Y | Y | Y | Y | Y | Y | Y | Y | Y | Y | Y | Y |
|  | (N) | N | N | N | N | Y | N | Y | N | Y | Y | N | N | Y | N | Y | Y | Y | N | Y | Y |
| Experimental animals | (O) | Y | Y | Y | Y | Y | N | Y | Y | Y | Y | Y | Y | Y | Y | Y | Y | Y | Y | Y | Y |
|  | (P) | N | N | N | N | N | N | N | N | N | N | N | N | N | N | N | N | Y | Y | Y | N |
| Experimental procedures | (Q) | Y | Y | Y | Y | Y | Y | Y | Y | Y | Y | Y | Y | Y | Y | Y | Y | Y | Y | Y | Y |
|  | (R) | Y | Y | Y | Y | Y | N | Y | Y | Y | Y | Y | Y | Y | Y | Y | Y | Y | Y | Y | Y |
|  | (S) | Y | Y | Y | Y | Y | N | Y | Y | Y | Y | Y | N | Y | Y | N | N | N | N | N | Y |
|  | (T) | Y | Y | N | Y | Y | N | Y | Y | Y | Y | Y | Y | Y | Y | Y | Y | Y | Y | Y | Y |
| Results | (U) | Y | Y | Y | Y | Y | Y | Y | Y | Y | Y | Y | Y | Y | Y | Y | Y | Y | Y | Y | Y |
|  | (V) | N | N | N | Y | N | N | N | N | N | N | N | N | N | Y | N | N | N | N | N | N |

(A): Provide brief details of study design including the groups being compared, including control groups; (B): Provide brief details of study design including the experimental unit; (C): Specify the exact number of experimental units allocated to each group, and the total number in each experiment.; (D): Explain how the sample size was decided.; (E): Describe any criteria used for including and excluding animals (or experimental units) during the experiment, and data points during the analysis.; (F): For each experimental group, report any animals, experimental units or data points not included in the analysis and explain why.; (G): For each analysis, report the exact value of n in each experimental group.; (H): State whether randomization was used to allocate experimental units to control and treatment groups.; (I): Describe the strategy used to minimize potential confounders such as the order of treatments and measurements, or animal/cage location.; (J): Describe who was aware of the group allocation at the different stages of the experiment.; (K): Clearly define all outcome measures assessed.; (L): For hypothesis-testing studies, specify the primary outcome measure.; (M): Provide details of the statistical methods used for each analysis.; (N): Describe any methods used to assess whether the data met the assumptions of the statistical approach, and what was done if the assumptions were not met.; (O): Provide species-appropriate details of the animals used.; (P): Provide further relevant information on the provenance of animals, health/immune status, genetic modification status, genotype, and any previous procedures.; (Q): Describe the procedures in enough detail including what was done, how it was done and what was used.; (R): Describe the procedures in enough detail including when and how often.; (S): Describe the procedures in enough detail including where (including detail of any acclimatization periods).; (T): Describe the procedures in enough detail including why (provide rationale for procedures).; (U): For each experiment conducted, including independent replications, report summary/descriptive statistics for each experimental group, with a measure of variability where applicable.; (V): For each experiment conducted, including independent replications, report if applicable, the effect size with a confidence interval.; Y: Yes; N: No.
